# A Novel Color Image Encryption Scheme Based on Hyperchaos and Hopfield Chaotic Neural Network

**DOI:** 10.3390/e24101474

**Published:** 2022-10-17

**Authors:** Yanan Wu, Jian Zeng, Wenjie Dong, Xinyu Li, Danyang Qin, Qun Ding

**Affiliations:** 1 Electronic Engineering College, Heilongjiang University, Harbin 150080, China; 2 Beijing Aerospace Institute of Automatic Control, Beijing 100854, China

**Keywords:** image encryption, hyperchaotic system, Hopfield chaotic neural network, DNA coding

## Abstract

Problems such as insufficient key space, lack of a one-time pad, and a simple encryption structure may emerge in existing encryption schemes. To solve these problems, and keep sensitive information safe, this paper proposes a plaintext-related color image encryption scheme. Firstly, a new five-dimensional hyperchaotic system is constructed in this paper, and its performance is analyzed. Secondly, this paper applies the Hopfield chaotic neural network together with the novel hyperchaotic system to propose a new encryption algorithm. The plaintext-related keys are generated by image chunking. The pseudo-random sequences iterated by the aforementioned systems are used as key streams. Therefore, the proposed pixel-level scrambling can be completed. Then the chaotic sequences are utilized to dynamically select the rules of DNA operations to complete the diffusion encryption. This paper also presents a series of security analyses of the proposed encryption scheme and compares it with other schemes to evaluate its performance. The results show that the key streams generated by the constructed hyperchaotic system and the Hopfield chaotic neural network improve the key space. The proposed encryption scheme provides a satisfying visual hiding result. Furthermore, it is resistant to a series of attacks and the problem of structural degradation caused by the simplicity of the encryption system’s structure.

## 1. Introduction

From the modification of hieroglyphics to post-quantum ciphers, cryptography has gradually taken shape and developed along with human civilization. With the development of technology, information has had a dramatic explosion. A great deal of privacy has been loaded onto the Internet. The development of tools such as streaming media and instant messaging has made it possible for social networks to connect countless individuals. Users of any status can share what they see online as they wish. Therefore, the security of various information carriers, especially digital images that carry abundant information, has received increasing attention.

Chaos is one of the major discoveries of the 20th century and its importance can be compared with relativity and quantum mechanics. Chaos is the unpredictable, pseudo-random motion exhibited by deterministic dynamical systems due to their sensitivity to initial values. The complex dynamical behavior in chaotic systems makes them widely applicable in communication, signal processing, and other fields. Compared with text, digital images are characterized by a larger information load, a stronger correlation of adjacent pixels, and higher redundancy. These characteristics lead to the unfitness of traditional encryption algorithms for image encryption [1]. Chaotic cryptography is a newly developing interdisciplinary science combining nonlinear science and cryptography. Researchers have taken advantage of chaotic systems in aspects such as pseudo-randomness, ergodicity, and utmost sensibility to initial values. These characteristics are beneficial for conducting efficient information hiding.

A low-dimensional chaotic system has the advantage of being simple to implement. Therefore, it is widely used in image encryption [2]. The low-dimensional chaotic system usually performs iteration to yield initial values of a high-dimensional chaotic system, and researchers have proposed schemes that combine low-dimensional chaos and high-dimensional chaos for encryption [3,4]. Some researchers also chose to improve on the existing low-dimensional chaotic systems to propose new chaotic mappings. Then, they applied the new mappings to encryption in combination with high-dimensional systems [5,6]. Existing experiments have shown that using high-dimensional chaotic systems for encryption can obtain a larger key space and improve the complexity of the algorithm. High-dimensional systems and multi-system cascades can achieve better encryption performance and also provide ideas for multi-image encryption [7,8,9]. In recent years, as DNA coding has advanced, it has gradually been used to implement image encryption in combination with chaotic systems. In [10], a new 4-D conservative hyperchaotic system was constructed. The authors conducted various evaluations of the chaotic system and the corresponding chaotic sequences. An image encryption scheme combining line-wise permutation with the DNA method in the process of diffusion was proposed. In [11], a chaotic-related image encryption algorithm composed of chunking permutation and DNA operations was proposed. Plaintext-related initial keys are yielded for the system iterations. The pseudo-random sequences are applied to shuffle pixels inside and between blocks. The pixel values are changed using DNA operations controlled by the sequences. In [12], a multidimensional image encryption scheme combining the DNA method and chaos was proposed. The authors utilized MD5 to collect the image features and then yield a user-related key. In this way, improvements to the traditional 3-D Lorenz system are made to construct a novel 4-D hyperchaotic Lorenz system. Then, plaintext images accomplish encoding with the DNA method. Most of the current DNA coding methods used in encryption choose three encoding rules of addition, subtraction, and XOR, while scholars have also designed some new DNA computing rules such as cycle shift [13].

Neural networks have been a popular topic in recent years. Aihara et al. [14] found rich nonlinear dynamical behaviors in neural networks in their research and creatively proposed the concept of chaotic neural networks. Chaotic neural networks possess associative memory and highly parallel properties of neural networks, as well as chaotic properties. Therefore, combining chaotic neural networks with cryptography can theoretically yield considerable encryption results. In 1982, the physicist Hopfield introduced the classical discrete Hopfield neural network model in [15]. Two years later, Hopfield designed a circuit to implement a continuous Hopfield neural network by simulating the connections between neurons through electronic circuits [16]. Modification based on the classical model of the Hopfield neural network is one of the main ideas for implementing chaotic neural networks. Combining chaos theory and neural networks to achieve secure and efficient image encryption is becoming a hot research topic in this field. A new chaos generator implementation using artificial neural networks was proposed by Ali et al. [17]. They use neural networks as the scrambling part of the chaos generator in image encryption systems to increase the cycle length while simultaneously avoiding the degradation problem of dynamical properties associated with the use of finite-dimensional spaces. The application of neural networks allows the chaotic sequence generator to have a larger key space. Liu et al. [18] applied the plaintext-dependent matrix generated by the Hopfield chaotic neural network to the second-round diffusion of the encryption process. This not only improves the sensitivity of the key but also makes it able to resist the common selective plaintext attack. Chaotic neural networks have also been widely used in the optimization of image encryption algorithms. Lakshmi et al. [19] proposed an encryption algorithm on the basis of Hopfield attractors without using other chaotic graphs. The results show suitable statistical properties and security, especially against the widely adopted chaotic graph attacks. In recent years, some researchers have launched studies on image encryption using Hopfield chaotic neural networks based on the chaotic properties of Hopfield neurons. Wang et al. [20] proposed a color image encryption algorithm based on Hopfield chaotic neural networks. Hu deciphered the CIEA-HCNN proposed by Wang et al. and pointed out that the chaotic pseudo-random sequences in this scheme are independent of the plaintext image. The scrambling–diffusion encryption structure will degenerate into a pure scrambling structure after the diffusion encryption structure with bit-XOR as the main operation is deciphered. The encryption structure is simple and cannot effectively resist the selective plaintext attack in a comprehensive view [21]. Tirdad et al. [22] used the Hopfield neural network as a pseudo-random number generator, but its randomness performed poorly. In terms of cross-integration with cryptography, Hopfield chaotic neural networks mostly act as chaotic sequence generators, and the randomness of the chaotic sequences they generate has been tested by NIST test suites in some recent papers [23], which showed that using Hopfield chaotic neural networks as sequence generators provides inspiration for the development of cryptography, but in terms of plaintext association, topology selection (related to randomness, sensitivity) and other aspects need to be improved. In addition, most encryption schemes use a combination of chaotic systems and DNA coding, and there are relatively few schemes combined with Hopfield neural networks. Moreover, chaotic systems and Hopfield chaotic neural networks can be implemented in hardware [24,25] that can be deployed to hardware platforms such as FPGAs and have the potential for a wide range of applications in engineering.

There are also some new directions, such as compressive sensing combined with DNA coding, compressive sensing combined with Hopfield chaotic neural networks for image encryption, quantum cryptography and DNA coding applied together in the design of encryption schemes, and memristive chaotic systems and DNA operations jointly applied in encryption [26,27,28,29,30]. These approaches can be applied to text, audio, and video encryption as well [31,32]. There are works concerned with cryptanalysis among encryption methods based on DNA operations. Researchers enhanced the scheme based on deciphering algorithms adopting the Feistel network and hyperchaotic system [33,34].

Based on the above, the main contributions of this paper are as follows:

(1) Considering the key space, this paper first constructs a novel 5-D hyperchaotic system, which is then combined with the existing 3-D Hopfield chaotic neural network to iteratively generate eight chaotic sequences, all of which are represented with double precision. Thus, a very large key space can be obtained to resist brute attacks.
(2) To obtain plaintext-related keys, this paper intends to generate the initial condition keys and the selection keys by image chunking. In the case of building a key table of all possible combinations, the selection key is used to select the condition key. Therefore, the initial conditions of the 5-D hyperchaotic system and the Hopfield chaotic neural network will be yielded. The scrambling matrix is generated according to the chaotic sequences to shuffle pixels of R, G, and B channels. That is, different images correspond to different keys and scrambling coordinates.
(3) A new image encryption scheme combining the hyperchaotic system and chaotic neural network is proposed. A simple structure of diffusion will lead to the degradation of the encryption system into a permutation-only structure. Therefore, this paper introduces DNA coding and dynamically selects the coding rules and computing rules through chaotic sequences to ensure the complexity of the encryption structure.

The paper is organized as follows: The basic methodology description of the proposed scheme is given in Section 2, including the new five-dimensional hyperchaotic system, Hopfield chaotic neural network, and DNA coding. Section 3 gives a detailed explanation of the proposed method, including pixel-level scrambling encryption, diffusion encryption combined with DNA operations, and chaotic sequences. In Section 4, the obtained results and the security analysis are discussed. At last, Section 5 gives the conclusion of this paper.

## 2. Preliminaries

### 2.1. A New 5-D Hyperchaotic System

**Figure 1:**
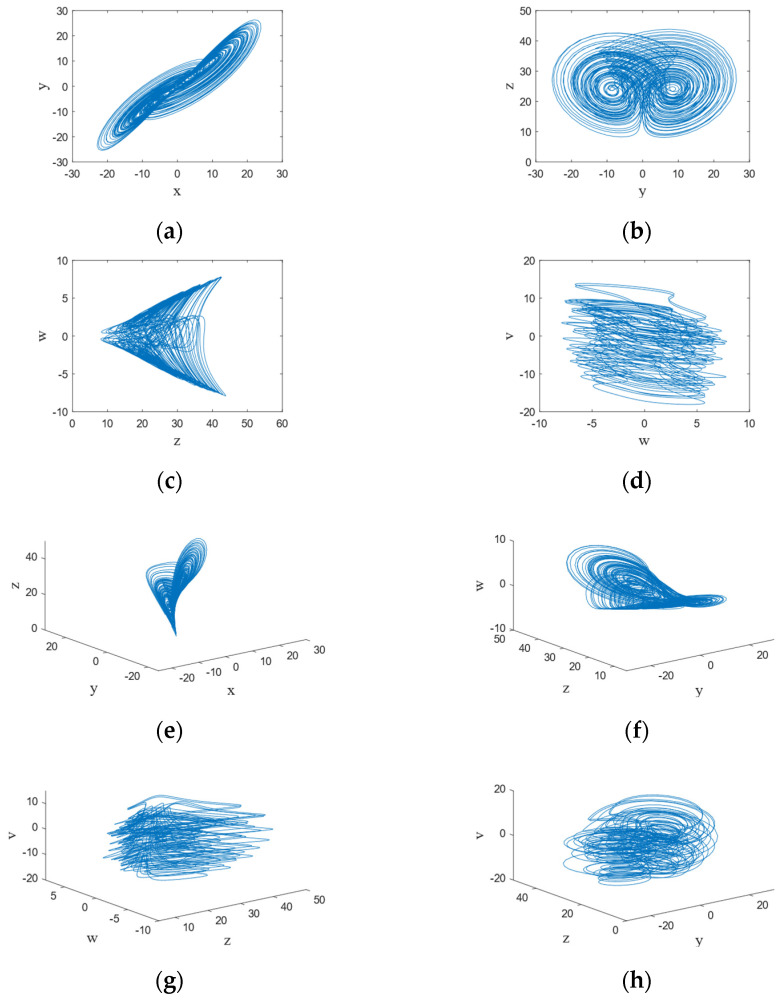
The hyperchaotic system’s phase diagrams: (**a**) x−y plane; (**b**) y−z plane; (**c**) z−w plane; (**d**) w−−v plane; (**e**) x−y−z plane; (**f**) y−z−w plane; (**g**) z−w−v plane; (**h**) y−z−v plane.

Chaotic phenomena are widely found in deterministic nonlinear systems with pseudo-random behavior and extreme sensitivity to initial value parameters. Although low-dimensional chaotic systems are widely used in image encryption systems in view of their simplicity of implementation and low computing complexity, high-dimensional chaotic systems present stronger nonlinear properties compared to low-dimensional chaotic systems and can achieve better encryption performance. Chaotic systems with two or more positive Lyapunov exponents are defined as hyperchaotic systems [35], implying better confidentiality, larger key space, and more complex unpredictable nonlinear behavior, which helps to generate keys with better randomness. Therefore, a new 5-D hyperchaotic system (HC5D) is constructed in this paper, and its state equation is shown in Equation (1):

(1) $$\left\{ \begin{array}{c} \dot{x}=-40x+40y+0.35w^{2}\\ \dot{y}=23.4y-xz-v\\ \dot{z}=xy-3z\\ \dot{w}=-0.2zy-10w\\ \dot{v}=cy \end{array}\right.$$

where x,y,z,w,v are the state variables of the proposed system and c is the control parameter. When the control parameter c is in the range of −0.9 to 41.5, the system exhibits chaotic behavior. The chaotic sequences and chaotic attractors generated by the proposed hyperchaotic system are shown in Figure 1. According to the following phase figures, the chaotic characteristics of the system can be observed.

**Figure 2:**
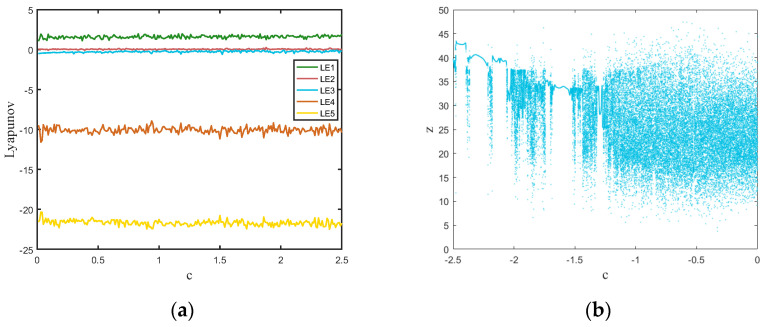
(**a**) Lyapunov exponent spectrum: c∈(0,2.5]; (**b**) bifurcation diagram: c∈(−2.5,0].

The Lyapunov exponents of the hyperchaotic system can be calculated as $LE_{1}=1.575$, $LE_{2}=0.142$, $LE_{3}=0.001$, $LE_{4}=-10.361$, and $LE_{5}=-21.736$. There are two positives, implying a hyperchaotic system. The Lyapunov calculation plot is shown in Figure 2a. The bifurcation diagram indicating a state transition from non-chaos into chaos with a 0.00005 step of c is shown in Figure 2b. It is demonstrated that the chaotic system exhibits a disorderly uniform distribution.

NIST can evaluate the randomness of data by providing a set of determination criteria. To guarantee the random performance of the generated chaotic sequences, the NIST SP 800-22 for the quantitative description of sequence randomness has been employed. The results are shown in Table 1. The data in the table show that the chaotic sequences successfully pass the test. That is, the chaotic sequences generated by the constructed HC5D in this paper are equipped with suitable randomness.

### 2.2. Hopfield Chaotic Neural Network

The Hopfield chaotic neural network (HCNN) is a single-layer fully interconnected feedback network with recurrent and recursive properties and has been adopted in secure communication and signal processing. The fully connected structure of HCNN introduces self-feedback, and recurrent neural networks produce constant state changes as the network is activated by the input due to the feedback from its output to its input. This topology is consistent with the neural feedback loops that are abundantly present in biological nervous systems. The classical HCNN can be modeled by Equation (2):

(2) $$\begin{array}{l} {\dot{x}}_{i}=-kx_{i}+Wf(x_{i})\\ f(x_{i})=\tanh(x_{i}) \end{array}$$

where $x_{i}$ is a column vector of the neuron state variable; k is the scale factor, which is usually taken as 1; and W is the weight matrix, and its elements $w_{ij}$ are the weights between $x_{i}$ and $x_{j}$, representing the strength of the connections between neurons. The activation function f is supposed to be a nonlinear continuous sigmoid-type function. The time-dependent hyperbolic tangent function is chosen as the activation to update the neuron states. The nonlinearity of the activation function is the origin of the nonlinear behavior of the neural network.

The feedback process continues until the network reaches a certain state. The network may present a steady state, a periodic state, or a chaotic state, and the key is to determine its weight coefficients, that is, the topology of the network. According to the literature [36], when W takes the value shown in Equation (3), the 3-D HCNN can be modeled by Equation (4):

(3) $$W=\left[\begin{array}{l} \;2\;\;\;-1.2\;\;0\\ 1.9+p\;1.71\;1.15\;\\ -4.75\;\;0\;\;1.1 \end{array}\right]$$

(4) $$\left[\begin{array}{l} {\dot{x}}_{1}\\ {\dot{x}}_{2}\\ {\dot{x}}_{3} \end{array}\right]=-\left[\begin{array}{l} x_{1}\\ x_{2}\\ x_{3} \end{array}\right]+W\left[\begin{array}{l} \tanh(x_{1})\\ \tanh(x_{2})\\ \tanh(x_{3}) \end{array}\right]$$

**Figure 3:**
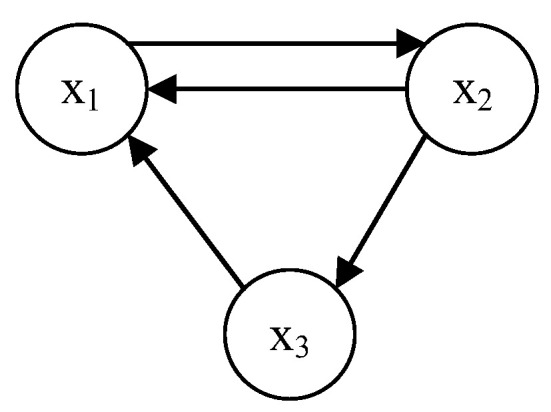
Topology of 3-D Hopfield chaotic neural network.

The corresponding neural network topology is shown in Figure 3.

The neural network exhibits satisfying chaotic properties when p=0.0997. The HCNN can be regarded as a complex chaotic mapping, and therefore it has the properties of chaos such as initial value sensitivity, pseudo-randomness, and ergodicity. These characteristics are inextricably linked to the principles of cryptography designed by Shannon in conjunction with the basic properties of chaos, “diffusion and confusion“ [37].

### 2.3. DNA Coding

DNA is composed of nucleotides, whose nucleobases are named adenine (A), cytosine (C), guanine (G), and thymine (T). Due to the natural mechanism, there is a basic complementarity theorem, where A is complementary to T and G is complementary to C [38]. The mechanism is similar to the complementarity of 1 and 0 in binary. The pixel values of grayscale images range from 0 to 255, which means the expression of an 8-bit binary number. According to the rules of DNA coding, eight valid coding rules are available to use in encryption, as shown in Table 2.

With the progress of DNA cryptology, some scholars have proposed algorithms such as addition and subtraction operations based on DNA sequences inspired by the basic principles of binary. In this paper, we use four common DNA computing rules, which are addition (+), subtraction (−), XOR (⊕), and XNOR (⊙), as shown in Table 3, Table 4, Table 5 and Table 6. The security of a single DNA coding or computing rule is low, and the security of the system can be further improved by controlling the dynamic selection of rules through chaotic sequences.

Therefore, the proposed scheme works by controlling the dynamic selection of eight DNA encoding and decoding rules and four computing rules through chaotic sequences generated by HC5D.

(5) $$w_{q}=\mathrm{floor}(\mathrm{mod}(w_{0}\times 10^{15},8))+1$$

(6) $$W_{0}=\mathrm{reshape}(w_{q},M,N)$$

(7) $$v_{q}=\mathrm{floor}(\mathrm{mod}(v_{0}\times 10^{15},4))+1$$

(8) $$V_{0}=\mathrm{reshape}(v_{q},M,4N)$$

where $w_{0}$ and $v_{0}$ are the chaotic sequences generated by the hyperchaotic system, and $w_{q}$ and $v_{q}$ are the quantized sequences. After quantization, the elements of $w_{q}$ will be integers in the range of [1,8], and elements of $v_{q}$ will be integers in the range of [1,4]. $W_{0}$ and $V_{0}$ are the matrices for reshaping the quantized sequences with the sizes of M×N and M×4N, respectively. $W_{0}$ is used to control the selection of DNA coding rules, and $V_{0}$ controls the selection of DNA computing rules.

## 3. The Proposed Scheme

### 3.1. Key Generation

This section proposes the method of plaintext-related key generation for resisting plaintext attacks, including the initial conditional key required by HC5D and HCNN and the selection key that controls the key-picking process.

**Figure 4:**
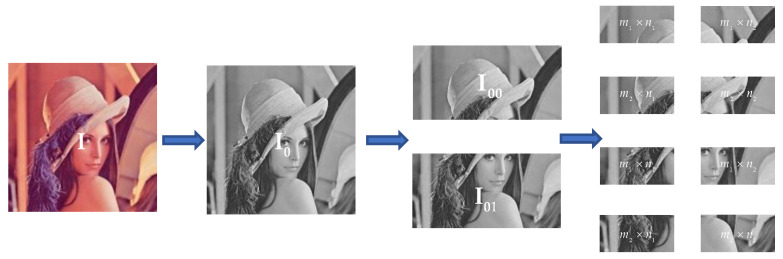
Image chunking demonstration.

For a plaintext image I of size M×N, expect to satisfy M≥4 and N≥2, and assume that M is an even number (if not, pad zero to the bottom row of the image matrix). Convert I into a grayscale image $I_{0}$ as shown in Figure 4 and then divide it into two parts to obtain the image $I_{00}$, $I_{01}$ of $M^{\prime }\times N$, where $M^{\prime }=M/2$. Then, the image is subdivided into eight independent blocks, and they will produce the initial condition keys, where $m_{1}=(M-\mathrm{mod}(M,4))/4$, $m_{2}=M^{\prime }-m_{1}$, $n_{1}=(N-\mathrm{mod}(N,2))/2$, $n_{2}=N-n_{1}$. Finally, the initial conditional key associated with the plaintext is generated according to Equation (9).

(9) $$k_{i}=\mathrm{double}(\sin(sum_{i}))$$

where i=1,2,⋯,8; $sum_{i}$ denotes the accumulation gray value of the i-th block, which works as the input of the sine function; and $k_{i}$ is defined as a double type.

The selection keys are generated by $I_{00}$, $I_{01}$. Since eight keys are generated above, five of them are needed for the initial conditions of the hyperchaotic system, and three are needed for the HCNN, so according to the knowledge related to permutation and combination, there are $A_{8}^{5}$ ways to combine keys for the hyperchaotic system and $A_{8}^{3}$ ways to combine keys for the HCNN. That is, there are 56 combinations.

Thus, we build two key tables to list all possible combinations. The sums of the pixels among the two image blocks are calculated separately. Then the index values $s_{1}$ and $s_{2}$ of the supposed combination are obtained according to Equation (10), which in turn generates the plaintext-related selection keys used to control the initial conditional keys’ combination.

(10) s=floor(mod(sum(I_pix),56))+1

where I_pix is the pixel value of $I_{00}$ and $I_{01}$. The range of s is 1 to 56.

### 3.2. Scrambling Process

This section proposes the image scrambling method controlled by chaotic sequences to effectively shuffle pixels’ positions.

The keys obtained in Section 3.1 are input into HC5D and HCNN, and the chaotic sequence is generated by iterating the systems. The horizontal coordinate X table and vertical coordinate Y table of the disordered pixels are constructed using the chaos matrix. By finding values in the X table and Y table, the new position of the pixel is determined, and thus the purpose of destroying the correlation of adjacent pixels is achieved. The specific steps are as follows:

**Step 1:**  The three channels of a color image Lena are separated according to Equation (11) to obtain the $I_{r}$, $I_{b}$, $I_{b}$ matrix of size M×N.
  (11) $$\left\{ \begin{array}{l} I_{r}=I(:,:,1)\\ I_{g}=I(:,:,2)\\ I_{b}=I(:,:,3) \end{array}\right.$$
**Step 2:**  The combination of the initial keys $\left(x_{00},y_{00},z_{00},w_{00},v_{00}\right)$ and $\left(x_{10},y_{10},z_{10}\right)$, which are used as the initial conditions for HC5D and HCNN, respectively, is obtained according to Section 3.1.
**Step 3:**  The initial conditions are input into HC5D and HCNN, and the chaotic sequences $x_{0}$, $y_{0}$, $z_{0}$, $w_{0}$, $v_{0}$ and $x_{1}$, $y_{1}$, $z_{1}$ are obtained by iteration according to Equations (1) and (2).
**Step 4:**  The former MN terms of the chaotic sequences $x_{1}$ and $x_{0}$ generated by the hyperchaotic system are reshaped using Equation (12), and the elements are sorted by column, while the row sorting of $y_{0}$ is performed. In this way, the values of x are in the range [1,M] and the values of y are in the range [1,N], so the coordinate table can be used as the index of the chaotic coordinates.
  (12) $$\left\{ \begin{array}{l} X=\mathrm{reshape}(x(1:MN),M,N)\\ \left[X_{up},X_{ind}]=\mathrm{sort}(X\right) \end{array}\right.$$

The new position matrix P can be obtained after obtaining the X table and Y-coordinate table, and the matrix P can be expressed by Equation (13):

(13) $$P(i,j)=(X_{ind}(i,j),Y_{ind}(i,j))$$

where i=1,2,⋯,M;j=1,2,⋯,N. $X_{ind}$, $Y_{ind}$ is a table of the generated horizontal and vertical coordinate indexes. After obtaining the position matrix, the R-channel of the image is scrambled.

**Step 5:**  The scrambling of the G channel is achieved by repeating Step 4 using the sequences $y_{0}$ and $y_{1}$ of length MN.
**Step 6:**  The B channel is scrambled using the sequences $y_{0}$ and $x_{1}$ of length MN, and Step 4 is repeated.

**Figure 5:**
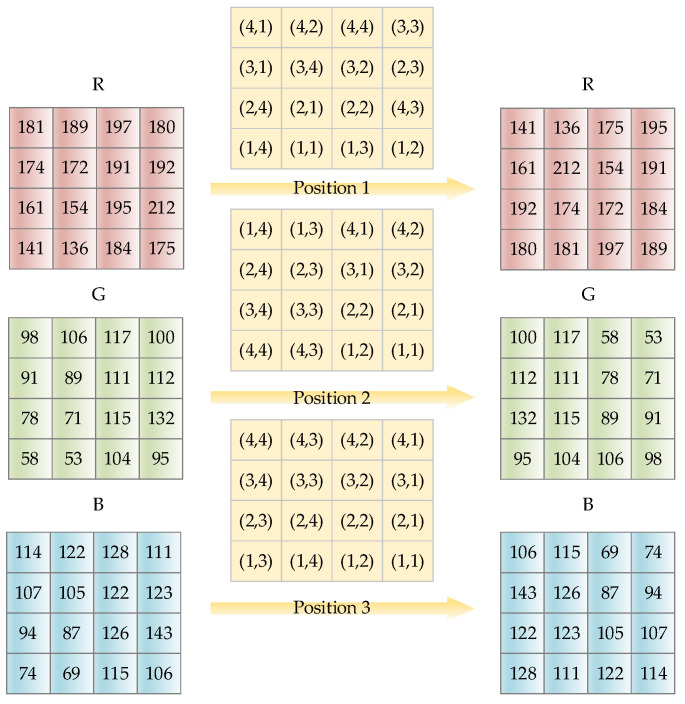
Example: demonstration of the proposed scrambling method for a 4 × 4 image.

The scrambling process is shown in Figure 5. Three channels are shuffled pixel by pixel with the control of the chaotic sequences, which are generated by HC5D and HCNN. Position 1, Position 2, and Position 3 are scrambling matrices P(i,j) composed of elements from quantized chaotic sequences. R, G, and B are channels of the image.

**Figure 6:**
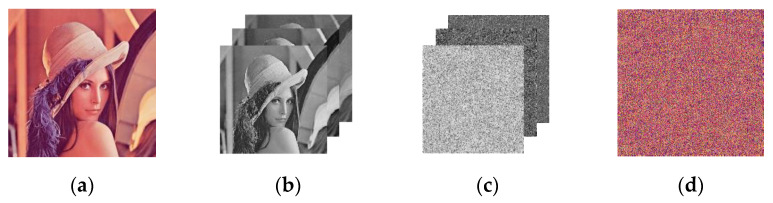
The result of the proposed scrambling method on Lena image: (**a**) the plaintext image; (**b**) the R, G, and B channels; (**c**) the scrambled R, G, and B channels for 1 round; (**d**) the scrambled encrypted image.

The scrambling effect is shown in Figure 6, and it can be seen that the proposed scrambling method requires only one round to obtain a visually satisfying hiding result. The scrambling coordinate matrix is also different for different images.

**Figure 7:**
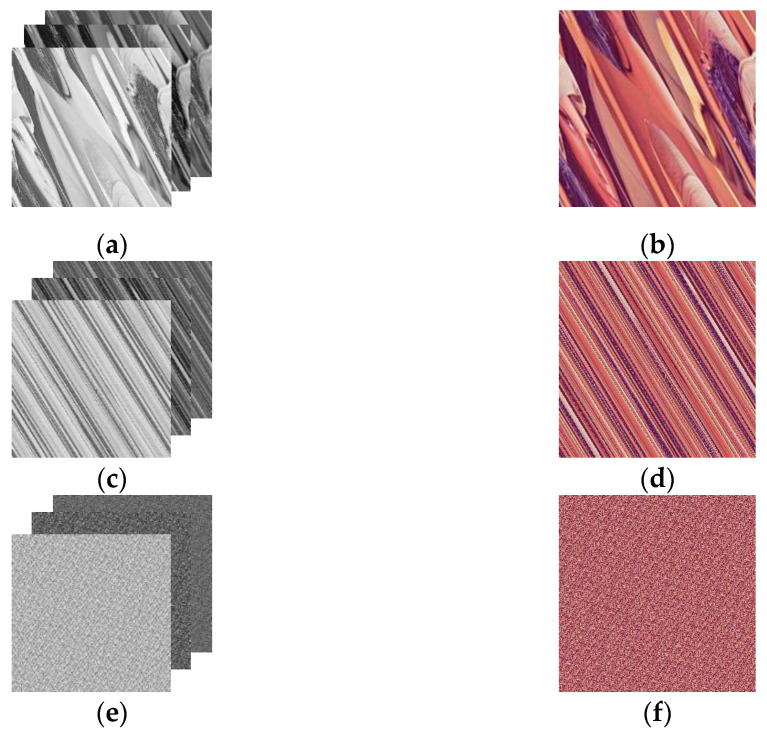
Lena with Arnold scrambling: (**a**) scrambled 1-round R, G, and B channels; (**b**) scrambled 1-round encrypted image; (**c**) scrambled 3-round R, G, and B channels; (**d**) scrambled 3-round encrypted image; (**e**) scrambled 6-round R, G, and B channels; (**f**) scrambled 6-round encrypted image.

The classical Arnold scrambling is restricted in image size. The unequal length and width of an image may lead to distortion. In addition, due to the mechanism of the Arnold algorithm, the scrambling is periodic with a transformation period of 60. Namely, the scrambling will obtain the original image after reaching the period. The following figures in Figure 7 show the effect of Arnold scrambling when *a* = 1, *b* = 1, and the round of scrambling *n* is 1, 3, and 6. It can be observed that the scrambling effect is unsatisfying at one round of scrambling. At three rounds, the image still shows obvious regularity. At six rounds, the image exhibits a relatively acceptable result but still shows tiny regularity.

### 3.3. Diffusion Process

The method in this section applies chaotic sequences generated by HC5D and HCNN to DNA coding. Then, it completes the diffusion encryption of images to change their pixel values. Moreover, compared with the general diffusion structure simply accomplished by bit-XOR, the method in this paper can effectively resist the problem of degradation of the encryption structure caused by the simplicity of the algorithm. The process of implementing this method is as follows:

**Step 1:**  The former MN terms of $w_{0}$ are quantized according to Equation (5) to obtain a sequence of integers $w_{0}$ with values of [1,8], and then the sequence is quantized and reshaped according to Equation (6) to obtain a control matrix $W_{0}(M,N)$ for dynamic selection of the DNA coding rules of the three-channel matrix.
**Step 2:**  According to Table 2, the three channels of the image are coded separately to obtain the coded matrix $D_{r0}$, $D_{g0}$, $D_{b0}$ of size M×4N.
**Step 3:**  Using Equation (14), the former MN terms of the key stream $z_{0}$, $z_{1}$, and the MN+1 to 2MN elements of $z_{0}$, are first quantized as integers in the range [0,255] and then reshaped into matrices $Z_{0}$, $Z_{1}$, $Z_{2}$ of size M×N.
  (14) $$\left\{ \begin{array}{l} z_{q}=\mathrm{floor}(\mathrm{mod}(z\times 10^{15},256))\\ Z=\mathrm{reshape}\left(z_{q},M,N\right) \end{array}\right.$$
  where z denotes the $z_{0}$ or $z_{1}$ sequence, $z_{q}$ denotes the quantized sequence, and Z denotes the matrix after a reorganization of the $z_{q}$ sequence. Equation (14) corresponds to extracting the 15 bits after the decimal point of the pseudo-random sequence and then transforming them to values within the grayscale pixel range.
**Step 4:**  Repeat Step 1 for the MN+1 to 2MN terms of $w_{0}$ to obtain the control matrix $W_{1}$ of $Z_{0}$, $Z_{1}$, $Z_{2}$ for the dynamic selection of DNA encoding rules. Repeat Step 2 to achieve the DNA encoding of the $Z_{0}$, $Z_{1}$, $Z_{2}$ matrices.
**Step 5:**  Select the former 4MN terms of $v_{0}$ for quantization according to Equation (7) to obtain a sequence of integers $v_{q}$ in the range [1,4]. Then, reshape the obtained vector into a matrix $V_{0}(M,4N)$ according to Equation (8) to obtain the control matrix $V_{0}$ for the dynamic selection of DNA computing rules. The corresponding DNA operations between $D_{r0}$, $D_{g0}$, $D_{b0}$ and $Z_{0}$, $Z_{1}$, $Z_{2}$ are implemented to obtain three new DNA matrices $D_{r1}$, $D_{g1}$, $D_{b1}$.
**Step 6:**  Transform the 2MN+1 to 4MN elements of $w_{0}$, $z_{0}$ into a vector of size 4MN and then modularize it to the range [1,8] according to Equation (5). The variant N in Equation (6) is substituted into 4N to shape the vector into a matrix of M×4N. Therefore, the control matrix $W_{2}(M,4N)$ for the dynamic selection of DNA decoding rules can be obtained.
**Step 7:**  DNA decoding is performed on $D_{r1}$, $D_{g1}$, $D_{b1}$, to obtain the single-channel matrices $C_{r}$, $C_{g}$, $C_{b}$ of size M×N, after diffusion encryption.

**Figure 8:**
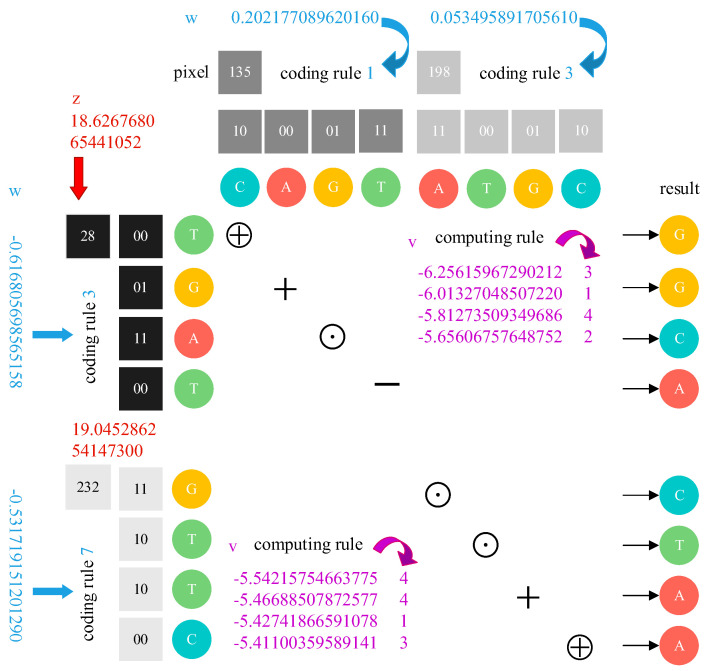
Example: demonstration of the DNA coding operation for two pixels with chaotic sequence elements.

Figure 8 demonstrates the basic process of DNA encoding and computing among pixels and chaotic sequences, taking two pixels as an example. The chaotic sequence w controls how the pixels and z sequence elements are encoded, chosen among eight rules according to Equations (5) and (6). Sequence v controls the corresponding computing rules, chosen among four rules according to Equations (7) and (8) in Section 2.3.

### 3.4. Encryption Scheme

**Figure 9:**
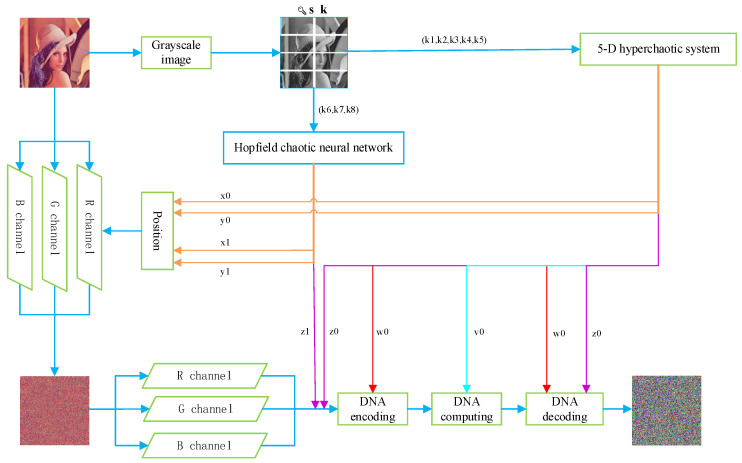
The encryption flowchart with Lena image as an example.

**Step 1:**  Input plaintext image I(M, N), according to the method described in Section 3.1, to obtain the grayscale image $I_{0}(M,\;N)$. Then, complete the chunking operation.
**Step 2:**  According to Equations (9) and (10), the initial conditional keys and the selection keys associated with the plaintext are updated.
**Step 3:**  Input the initial keys and iterate Equations (1) and (4) D+L times each, where L=4MN. To avoid the transient effect and to ensure random performance, the previous values are discarded to obtain the $x_{0}$, $y_{0}$, $z_{0}$, $w_{0}$, $v_{0}$, $x_{1}$, $y_{1}$, and $z_{1}$ key streams described in Section 3.1.
**Step 4:**  For the plaintext image I(M,N), separate its R, G, and B channels according to Equation (11) to obtain three images, $I_{r}$, $I_{g}$, and $I_{b}$, and take the former MN terms of $x_{0}$, $y_{0}$, $x_{1}$, and $y_{1}$ and the MN+1 to 2MN terms of $x_{0}$ and $y_{1}$ to obtain the combination of $\left(x_{0},y_{0}\right)$, $\left(x_{1},y_{1}\right)$, and $\left(x_{0},y_{1}\right)$. According to Equations (12) and (13), we can obtain the permutation matrix.
**Step 5:**  According to the method described in Section 3.2, the pixel-level scrambling is performed on $I_{r}$, $I_{g}$, and $I_{b}$, and the scrambled three channels are obtained simultaneously. Combine them to yield the scrambled image.
**Step 6:**  $w_{0}$, $v_{0}$, $z_{0}$, and $z_{1}$ are quantized and recombined according to the scheme designed in Section 3.3 to obtain the encoding matrices $W_{0}$ and $W_{1}$; the decoding matrix $W_{2}$; the $V_{0}$ matrix, which controls the computing rules; and the $Z_{1}$ and $Z_{2}$ matrices, which compute with the DNA images. The diffusion encryption of the three channels of the permuted image is completed. The three channels are merged to gain the cipher image C(M,N). The decryption process is the reverse process of encryption $Z_{0}$.
**Step 7:**  The complete encryption flow is shown in Figure 9.

**Figure 10:**
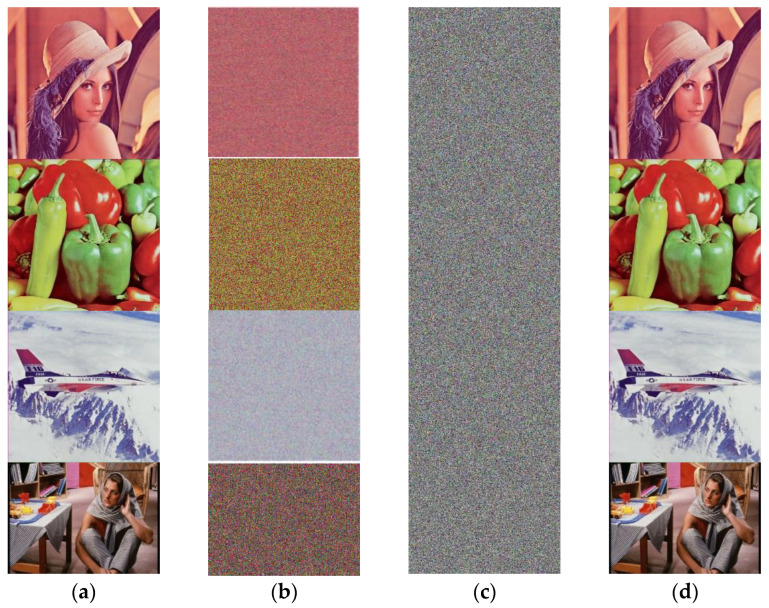
The results of the proposed scheme: (**a**) the plaintext images; (**b**) the permuted images; (**c**) the ciphertext images; (**d**) the decrypted images.

The encryption results are shown in Figure 10. It can be seen that all the ciphertext images are evenly distributed like snowflakes, and no meaningful information can be obtained visually. The results show that the proposed scheme can successfully hide the plaintext image as well as obtain a satisfying encryption effect. In this paper, by processing the image pixel by pixel, the time complexity calculation is M × N for an image of size M × N. Considering the DNA operation in diffusion, the coefficient should be 4. Calculating the asymptotic time complexity without considering the constant term, the complexity of the algorithm can be obtained as O (M × N); that is, the complexity keeps a linear relationship with the input image size. Furthermore, taking Lena (512 × 512) as an example, the running time is 2.5182 s, as a result of the trade-off between complexity and running time.

## 4. Security Analysis of the Scheme

In this section, we evaluate the performance of the proposed scheme through comprehensive software simulations. All tests were performed on the MATLAB R2016a platform using a computer equipped with an Intel i7-10875H processor with a CPU of 2.30 GHz and memory of 16 GB.

### 4.1. Key Analysis

#### 4.1.1. Key Space

In this paper, the key space consists of initial condition keys $k_{1},k_{2},\cdots ,k_{8}$ yielded by eight chunking blocks and selection keys $s_{1},s_{2}$ yielded by two chunking blocks, from the plaintext image. As mentioned before, eight initial keys are represented as double precision types. That is, the computation precision is $10^{-15}$ for each initial condition key. While the data type of $s_{1},s_{2}$ is fixed-point, the key space excluding $s_{1},s_{2}$ can be calculated as $10^{15}\times 10^{15}\times 10^{15}\times 10^{15}\times 10^{15}\times 10^{15}\times 10^{15}\times 10^{15}=10^{120}\approx 2^{399}$ at least. It can be seen from Table 7 that the key space of the proposed scheme is larger than the threshold $2^{128}$ and that of some of the other schemes. It is indicated that the key space benefiting from multiple systems is adequately large to defend against brute attacks as well.

#### 4.1.2. Key Sensitivity

**Figure 11:**
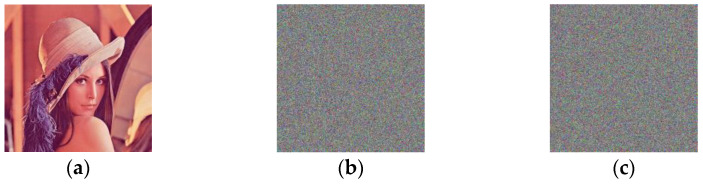
Key sensitivity test results: (**a**) the plaintext image; (**b**) the wrongly encrypted image; (**c**) the wrongly decrypted image.

When the key changes slightly, the corresponding encrypted and decrypted results exhibit radical changes due to the sensitivity of the key. This kind of phenomenon is what we expect in insecure communication channels in defense against eavesdroppers’ attacks. Figure 11a shows the plaintext image, (b) shows the image encrypted with the incorrect key with one decimal point change, and (c) shows the image decrypted with the incorrect key with one decimal point change. It is implied that a tiny modification of the key results in completely different corresponding images. Therefore, the key sensitivity of the system allows resistance to the modified key attack.

### 4.2. Statistical Analysis

#### 4.2.1. Gray Histogram

**Figure 12:**
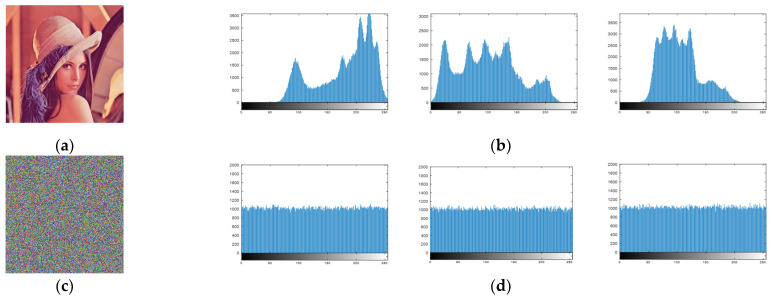
Histogram tests of the Lena image: (**a**) the plaintext image; (**b**) the histograms of R, G, and B channels of the original image; (**c**) the encrypted image; (**d**) the histograms of R, G, and B channels of the encrypted image.

Significant fluctuations in the histogram distribution of an encrypted image indicate that it cannot resist ciphertext attacks. Therefore, we conduct a gray histogram analysis of the proposed scheme to verify the quality of the encryption. A comparison of the histograms of the R, G, and B channels of the Lena image and the encrypted Lena image is shown in Figure 12. It can be visually seen that the channels of the original Lena image exhibit sharp fluctuations, while the ciphertext’s channels are uniformly distributed, leading to better performance in face of deciphering. Therefore, the system is highly resistant to statistical attacks for the provided experimental analysis.

#### 4.2.2. Correlation Analysis of Adjacent Pixels

Considering what is already known, an image as an information carrier is characterized by evident relevance between neighboring pixels. Therefore, eavesdroppers usually try to exploit this correlation to decipher encrypted images. This is why an encryption system should guarantee that the relevance of neighboring pixels is weakened as much as possible. In an encrypted image, the relevance among neighboring pixels is supposed to be close to zero as an ideal criterion. In this condition, it will be tough to infer the plaintext image while intercepting an encrypted image from an unsafe communication channel, such as the situation in satellite communications. The relevant equation for the neighboring pixels is defined as

(15) $$E(x)=\frac{1}{N}\sum_{i=1}^{N}x_{i}$$

(16) $$D(x)=\frac{1}{N}\sum_{i=1}^{N}{\left(x_{i}-E(x)\right)}^{2}$$

(17) $$\mathrm{Cov}(x,y)=\frac{1}{N}\sum_{i=1}^{N}\left(x_{i}-E(x)\right)\left(y_{i}-E(y)\right)$$

(18) $$r_{xy}=\frac{\left|\mathrm{Cov}(x,y)\right|}{\sqrt{D(x)\times D(y)}}$$

where $r_{xy}$ represents the correlation coefficient of neighboring pixels x and y. E(x) denotes the expectation. D(x) denotes the variance. Cov(x,y) denotes the covariance of x and y.

**Figure 13:**
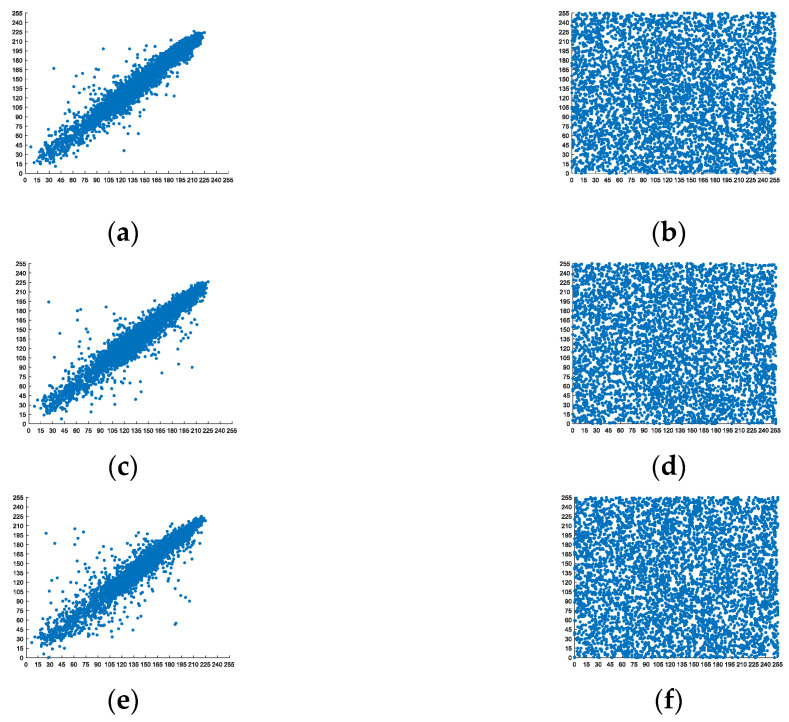
Pixel distributions of the Lena image: (**a**) The distribution of the original image in the horizontal direction. (**b**) The distribution of the encrypted image in the horizontal direction. (**c**) The distribution of the original image in the vertical direction. (**d**) The distribution of the encrypted image in the vertical direction. (**e**) The distribution of the original image in the diagonal direction. (**f**) The distribution of the encrypted image in the diagonal direction.

Figure 13 displays the pixel distributions of the original and encrypted Lena image. Figure 13a shows that the adjacent pixels in the horizontal direction of the original image cluster relatively densely around the diagonal of the figure. On the other hand, the pixels of the ciphertext image erratically scatter throughout the figure. It is implied that the proposed encryption method has efficiently reduced the relevance of the adjacent pixels of the image. The distributions in horizontal and diagonal directions are also consistent with the above description.

Meanwhile, a quantitative assessment has been conducted. The correlation metrics of three original images and relative encrypted images in three directions are presented in Table 8. It can be inferred from the data below that the applied encryption scheme has gained satisfying performance. The correlation comparison between the proposed scheme and other reference schemes is shown in Table 9. The proposed scheme achieved better performance overall, as we can see. That is, the proposed scheme is equipped with a better ability to resist statistical attacks, compared with the others.

#### 4.2.3. Information Entropy

Information entropy is derived from the concept of entropy in thermodynamics. This metric describes the average information after eliminating redundancy. For images, it provides a quantitative assessment of cluttered pixels. The ideal situation is that the entropy is close to 8, implying a uniform distribution of the image to resist statistical attacks. It is mathematically described as

(19) $$H=-\sum_{i=0}^{L}p(i)\log_{2}p(i)$$

where p(i) is the occurrence probability of the i-th pixel from the L-level gray image. The information entropy of an image is proportional to its unpredictability. The entropy details of the R, G, and B channels of before- and after-encryption images are listed in Table 10. It is shown that the values quite closely approach the theoretical value. Furthermore, the comparison of the proposed scheme with other schemes has been conducted on three channels, as shown in Table 11, proving a better performance of the proposed scheme.

To further verify the randomness of the ciphertext images, we used local Shannon entropy (LSE) to enhance the experiments. LSE can be calculated by Equation (20)

(20) $$\bar{H_{k,T_{B}}}(S)=\sum_{i=1}^{k}\frac{H(S_{i})}{k}$$

where k is the quantity of non-overlapping chunking blocks $S_{i}$; $T_{B}$ denotes the pixel quantity of every $S_{i}$. $H(S_{i})$ can be calculated by Equation (19). When k=25 and $T_{B}=1936$, the result of the LSE test performed on Lena (256 × 256) is 7.902654720. According to [44,45], this value passed the LSE test, validating the randomness of the ciphertext.

### 4.3. Classical Types of Attack

#### 4.3.1. Differential Attack

To be resistant to differential attacks related to the plaintext sensitivity, a cryptosystem should guarantee that tiny modifications in the plaintext image result in a significant difference in the ciphertext image. The number of pixels with change rate (NPCR) is one of the common measurement metrics, and the uniform average change rate intensity (UACI) is another one. Security criteria are met when the NPCR is close to the ideal value of 99.6094% and the UACI is close to 33.4635%. NPCR and UACI are described as

(21) $$NPCR=\frac{\sum_{i=1}^{M}\sum_{j=1}^{N}D(i,j)}{M\times N}\times 100\%$$

(22) $$UACI=\frac{\sum_{i=1}^{M}\sum_{j=1}^{N}\left|P_{1}(i,j)-P_{2}(i,j)\right|}{255\times M\times N}\times 100\%$$

where the size of the image is denoted as M×N. D(i,j) is the pixel difference between $P_{1}(i,j)$ and $P_{2}(i,j)$, defined as

(23) $$D(i,j)=\{\begin{array}{ll} 0 & P_{1}(i,j)=P_{2}(i,j)\\ 1 & P_{1}(i,j)\neq P_{2}(i,j) \end{array}$$

Table 12 gives the NPCR and UACI collections of the proposed scheme. Table 13 compares them with values from other references. The results show that the values of NPCR and UACI of the scheme are close to the ideal parameters, suggesting that the scheme can resist differential attacks better.

#### 4.3.2. Known and Chosen Plaintext Attack

Considering that the eavesdropper intercepts the plaintext and the ciphertext image, this leads to the eavesdropper guessing the key based on the difference while making tiny changes. In the proposed scheme, the key generation is determined by the images needed to be encrypted; that is, a one-time pad mechanism is applied as the input changing mechanism. Moreover, the designed scrambling method is associated with the key streams, and different key streams imply different shuffling position matrices for scrambling pixels. Based on the above analysis, the proposed scheme can resist plaintext-relative attacks.

### 4.4. Robustness Analysis

**Figure 14:**
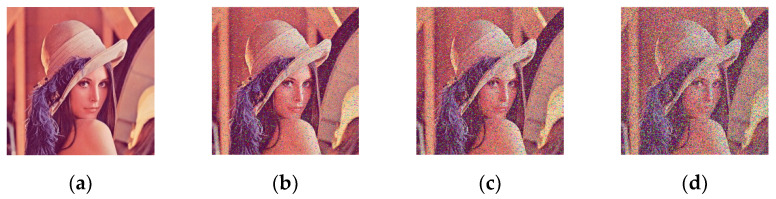
Experimental results of adding noise: (**a**) decrypted Lena image with the noise of density 0; (**b**) decrypted Lena image with the noise of density 0.05; (**c**) decrypted Lena image with the noise of density 0.1; (**d**) decrypted Lena image with the noise of density 0.2.

During communication, potential noise pollution of information exists in the transformation process. To assess the robustness of the proposed encryption system, tests of using different densities of noise to pollute the encrypted image were conducted separately. Figure 14 displays the decryption results of adding noise of densities 0, 0.05, 0.1, and 0.2. Although there are some snowflakes on the decrypted image, we can still distinguish valid information from the results. It is proved that the system is equipped with robustness against noise attacks.

## 5. Conclusions

In this paper, a new 5-D hyperchaotic system is constructed and a novel plaintext-correlated image encryption scheme based on the combination of the 5-D hyperchaotic system and the Hopfield chaotic neural network is proposed. Structurally, the scheme consists of two main encryption stages, perpetuation and diffusion. First, the original image is used for chunking to yield the initial condition keys and the selection keys for the initial key combination. Then, the initial keys are used to yield chaotic sequences of the two systems as key streams for the encryption system. Afterward, the key streams are used to construct the shuffling position matrices to complete the pixel-level scrambling. Finally, in the diffusion phase, the chaotic sequences and DNA coding are combined to achieve diffusion encryption. The R, G, and B channels are merged to obtain the complete encrypted image. Generally, the application of HC5D and HCNN introduces a huge key space, making the scheme effectively defendable against brute attacks. In addition, the system introduces a plaintext-relative key generation mechanism; due to the sensitivity to images, it is capable of defending against plaintext-relative attacks. Moreover, compared with the traditional cryptosystem based on a single chaotic system, combining HC5D with HCNN can obtain a more sophisticated encryption structure, avoiding the encryption system degradation problem caused by a simple structure. Thus, the encryption system can achieve higher security. Security analyses are carried out to validate the performance of the proposed scheme. In conclusion, the above statistics indicate that the proposed scheme can safeguard sensitive information and is attack-resistant.

## Figures and Tables

**Table 1:** Results of the NIST randomness test for the proposed hyperchaotic system.

Test	*p*-Value	Result
Approximate Entropy	0.933528	Pass
Block Frequency	0.557129	Pass
Cumulative Sum 1	0.974025	Pass
Cumulative Sum 2	0.974025	Pass
FFT	0.639925	Pass
Frequency	0.818524	Pass
Linear Complexity	0.889224	Pass
Longest Run	0.243165	Pass
Nonoverlapping Template	0.326447	Pass
Overlapping Template	0.326447	Pass
Random Excursion	0.416631	Pass
Random Excursions Variant	0.446108	Pass
Rank	0.867239	Pass
Runs	0.363268	Pass
Serial 1	0.324486	Pass
Serial 2	0.181049	Pass
Universal	0.125123	Pass

**Table 2:** DNA coding rules.

Rule	1	2	3	4	5	6	7	8
00	A	A	T	T	G	G	C	C
01	G	C	G	C	A	T	A	T
10	C	G	C	G	T	A	T	A
11	T	T	A	A	C	C	G	G

**Table 3:** DNA addition rules.

+	A	G	C	T
A	A	G	C	T
G	G	C	T	A
C	C	T	A	G
T	T	A	G	C

**Table 4:** DNA subtraction rules.

−	A	G	C	T
A	A	T	C	G
G	G	A	T	C
C	C	G	A	T
T	T	C	G	A

**Table 5:** DNA XOR rules.

⊕	A	G	C	T
A	A	G	C	T
G	G	A	T	C
C	C	T	A	G
T	T	C	G	A

**Table 6:** DNA XNOR rules.

	A	G	C	T
A	T	C	G	A
G	C	T	A	G
⊙ C	G	A	T	C
T	A	G	C	T

**Table 7:** Key space of the proposed scheme compared with other schemes from the literature.

Reference	Key Space
Ours	$10^{120}$
[3]	$2^{250}$
[39]	$6\times 2^{192}$
[40]	$10^{89}$
[41]	$2^{260}$

**Table 8:** Relevance of the adjacent pixels: tests of the plaintext images and ciphertext images.

Image		Horizontal		Vertical		Diagonal	
		Plaintext	Ciphertext	Plaintext	Ciphertext	Plaintext	Ciphertext
Lena	R	0.9756	0.0221	0.9864	0.0299	0.9639	−0.0120
	G	0.9755	0.0017	0.9875	0.0001	0.9650	0.0265
	B	0.9537	0.0073	0.9721	0.0116	0.9341	0.0077
Peppers	R	0.9625	−0.0123	0.9692	0.0077	0.9574	−0.0061
	G	0.9799	−0.0119	0.9832	0.0110	0.9675	0.0011
	B	0.9650	−0.0169	0.9609	−0.0201	0.9411	0.0244
Airplane	R	0.9717	0.0171	0.9515	−0.0075	0.9270	0.0011
	G	0.9538	0.0049	0.9670	−0.0136	0.9270	0.0218
	B	0.9619	0.0107	0.9311	0.0011	0.9102	0.0095

**Table 9:** The relevance of the encrypted Lena image compared with other references.

Reference	Horizontal	Vertical	Diagonal
Ours	0.0037	0.0139	0.0074
[39]	0.0076	–0.0125	0.0101
[40]	0.0214	0.0465	−0.0090
[42]	0.0139	0.0073	0.0104

**Table 10:** Information entropy tests of the plaintext images and ciphertext images.

Image		Entropy	
		Plaintext	Ciphertext
Lena (256 × 256)	R	7.2682	7.9994
	G	7.5901	7.9993
	B	6.9951	7.9992
Peppers (512 × 512)	R	7.3388	7.9994
	G	7.4963	7.9994
	B	7.0583	7.9992
Airplane (512 × 512)	R	6.7113	7.9993
	G	6.7853	7.9992
	B	6.2128	7.9993

**Table 11:** The entropy comparison of the encrypted Lena image (512 × 512) with other references.

Reference	Entropy		
	R	G	B
Ours	7.9993	7.9993	7.9992
[39]	7.9997	7.9937	7.9976
[41]	7.9991	7.9993	7.9993
[43]	7.9914	7.9907	7.9907

**Table 12:** The NPCR and UACI data of different images.

Image	NPCR (%)	UACI (%)
Lena	99.6081	33.4478
Peppers	99.6113	33.4462
Airplane	99.6156	33.4521
Barbara	99.6014	33.4496

**Table 13:** The NPCR and UACI results compared with other referenced literature.

Reference	NPCR (%)	UACI (%)
Ours	99.6081	33.4478
[41]	99.6403	33.4968
[43]	99.6211	33.5113
[46]	99.6098	33.4477

## Data Availability

Not applicable.
